# Genetic and Common Environmental Contributions to Familial Resemblances in Plasma Carotenoid Concentrations in Healthy Families

**DOI:** 10.3390/nu10081002

**Published:** 2018-07-31

**Authors:** Bénédicte L. Tremblay, Frédéric Guénard, Benoît Lamarche, Louis Pérusse, Marie-Claude Vohl

**Affiliations:** Institute of Nutrition and Functional Foods (INAF), Laval University, 2440 Hochelaga Blvd, Quebec City, QC G1V 0A6, Canada; benedicte-l.tremblay.1@ulaval.ca (B.L.T.); frederic.guenard@fsaa.ulaval.ca (F.G.); benoit.lamarche@fsaa.ulaval.ca (B.L.); louis.perusse@kin.ulaval.ca (L.P.)

**Keywords:** β-cryptoxanthin, bivariate analysis, cardiometabolic risk factors, carotenoids, familial resemblances, French Canadians, heritability, lutein, lycopene

## Abstract

Carotenoids have shown an interindividual variability that may be due to genetic factors. The only study that has reported heritability of serum α- and β-carotene has not considered the environmental component. This study aimed to estimate the contribution of both genetic and common environmental effects to the variance of carotenoid concentrations and to test whether their phenotypic correlations with cardiometabolic risk factors are explained by shared genetic and environmental effects. Plasma carotenoid concentrations (α-carotene, β-carotene, β-cryptoxanthin, lutein, lycopene, zeaxanthin, and total carotenoids) of 48 healthy subjects were measured. Heritability estimates of carotenoid concentrations were calculated using the variance component method. Lutein and lycopene showed a significant familial effect (*p* = 6 × 10^−6^ and 0.0043, respectively). Maximal heritability, genetic heritability, and common environmental effect were computed for lutein (88.3%, 43.8%, and 44.5%, respectively) and lycopene (45.2%, 0%, and 45.2%, respectively). Significant phenotypic correlations between carotenoid concentrations and cardiometabolic risk factors were obtained for β-cryptoxanthin, lycopene, and zeaxanthin. Familial resemblances in lycopene concentrations were mainly attributable to common environmental effects, while for lutein concentrations they were attributable to genetic and common environmental effects. Common genetic and environmental factors may influence carotenoids and cardiometabolic risk factors, but further studies are needed to better understand the potential impact on disease development.

## 1. Introduction

Adoption of healthy lifestyle habits, including healthy eating, is the cornerstone of chronic disease prevention [1,2]. Fruit and vegetable (FAV) consumption is inversely associated with chronic diseases [3,4]. Methods used to evaluate food consumption, such as 24 h recalls, food frequency questionnaires, and food diaries rely on self-reported data, which can distort estimation of certain food intakes [5,6]. Although strategies have been developed to better manage randomness and mitigate systematic errors, it is not possible to completely eliminate bias from self-reporting dietary assessment methods [7].

In that regard, measuring biomarkers of food consumption in blood, urine, and tissue may represent a more objective method to evaluate dietary intakes and patterns [8,9]. Carotenoids are a reliable biomarker of FAV consumption [10,11,12]. Indeed, FAV provide over 90% of daily carotenoid intake [13]. Carotenoids are a family of more than 700 fat-soluble pigments, but α-carotene, β-carotene, β-cryptoxanthin, lutein, lycopene, and zeaxanthin represent over 95% of total circulating carotenoids in human plasma or serum [13,14].

Interindividual variability in circulating carotenoids has been observed and may be partly attributable to genetic factors that cause differences in the absorption, assimilation, distribution, metabolism, and excretion of carotenoids [15,16,17]. Moreover, several genome-wide associations studies have identified genetic variants that influence circulating carotenoid concentrations [15,18,19,20]. Until now, only one study has reported heritability of serum carotenoids. The SAFARI study investigated genetics of serum carotenoids in Mexican-American children [21]. More specifically, it reported high genetic heritability of α-carotene and β-carotene along with phenotypic and genetic correlations of α-carotene and β-carotene with obesity-related traits [21]. However, it did not account for the environmental component in its heritability estimates [21]. 

The aim of the present study was to estimate the contribution of both genetic and common environmental effects to the variance of plasma carotenoid concentrations (α-carotene, β-carotene, β-cryptoxanthin, lutein, lycopene, zeaxanthin, and total carotenoids) and to test whether their phenotypic correlations with traditional cardiometabolic (CM) risk factors could be explained by shared genetic and environmental effects. Thus, we tested the hypothesis that both genetic and common environmental effects contribute to the variance of plasma carotenoid concentrations and that shared genetic and environmental effects explain their phenotypic correlations with CM risk factors. To test this hypothesis, the variance component method and bivariate genetic analysis were performed on a family-based sample of 48 French Canadians from 16 families. Our results highlighted that familial resemblances in lycopene concentrations were mainly attributable to common environmental effects, while for lutein concentrations they were attributable to both genetic and common environmental effects. Common genetic and environmental factors seem to influence carotenoids and CM risk factors but further studies are needed to better understand these possible influences and their potential impact on disease development.

## 2. Materials and Methods

### 2.1. Patients and Design

A total of 48 Caucasian French-Canadian subjects from 16 families were recruited in the Greater Quebec City metropolitan area, in Canada, as part of the GENERATION Study, for which recruitment began in May 2011. The GENERATION Study was designed to evaluate familial resemblances in omics (DNA methylation [22] and gene expression [23]) and metabolic (metabolites and carotenoids) profiles in healthy families. Families were composed of 16 mothers, 6 fathers, and 26 children. The majority of parents had a university education and a family income >80,000$ CAN. Inclusion criteria were that families living under the same roof comprise at least the mother and one child aged between 8 and 18. Parents had to be the biological parents of their child (or children), in good general health, with body mass index (BMI) ranging between 18 and 35 kg/m^2^. The use of Synthroid^®^ (AbbVie Inc. North Chicago, IL, USA) (levothyroxine) or oral contraceptive was tolerated. Children also had to be in good general health. Exclusion criteria were smoking, self-reported history of metabolic conditions requiring treatment, and use of psychostimulators [Ritalin^®^ (Novartis Pharmaceuticals Corporation, East Hanover, NJ, USA) (methylphenidate), Concerta^®^ (Janssen Pharmaceuticals, Inc. Raritan, NJ, USA) (methylphenidate), and Strattera^®^ (Eli Lilly and Company, IN, USA) (atomoxetine)]. Parents and children were asked to complete several dietary, physical activity, medical history, and pregnancy questionnaires under the supervision of a registered dietitian during their visit at the Institute of Nutrition and Functional Foods (INAF). The experimental protocol was approved by the Ethics Committees of Laval University Hospital Research Center and Laval University. All participants (adults and children) signed an informed consent document. Parental consent was also obtained by signing the child consent document.

### 2.2. Anthropometric and Cardiometabolic Measurements

Body weight, waist girth, and height were measured according to the procedures recommended by the Airlie Conference [24]. Blood samples were collected from an antecubital vein into vacutainer tubes containing ethylenediaminetetra-acetic acid (EDTA) after a 12 h overnight fast and 48 h alcohol abstinence. Plasma was separated by centrifugation (2500 g for 10 min at 4 °C), and samples were aliquoted and frozen (–80 °C) for subsequent analyses. Enzymatic assays were used to measure plasma total cholesterol (TC) and triglyceride (TG) concentrations [25,26]. Precipitation of very low-density lipoprotein (VLDL) and low-density lipoprotein (LDL) particles in the infranatant with heparin manganese chloride generated the high-density lipoprotein cholesterol (HDL-C) fraction [27]. LDL cholesterol (LDL-C) was calculated with the Friedewald formula [28]. Fasting glucose levels were enzymatically measured [29]. Using a sensitive assay, plasma C-reactive protein (CRP) was measured by nephelometry (Behring Latex-Enhanced on the Behring Nephelometer BN-100; Prospec equipment, Behring Diagnostic, Westwood, MA, USA) [30]. 

### 2.3. Carotenoid Measurements

Carotenoid standards were purchased from Sigma (Oakville, ON, Canada). Stock solutions for each carotenoid were prepared (1 mg in 100 mL of solvent) in ethanol for β-cryptoxanthin, lutein, and zeaxanthin and in hexane for β-carotene and lycopene. The concentration of each stock solution was determined using an ultraviolet (UV) spectrophotometer and the specific molecular extinction coefficient of the molecule [31]. Carotenoid standards were solubilized with methanol/dichloromethane (65/35, *v*/*v*) for a final concentration of 2 μM. These solutions were then used to perform calibration curves. Retinol acetate (15 μM) was used as an internal standard. 

Plasma samples were thawed a few hours before analysis. A total of 100 μL of plasma, 20 μL of 2-propanol, and 20 μL of internal standard were transferred in Eppendorf tubes. Samples were transferred on a 400 μL fixed well plate (ISOLUTE^®^ SLE+, Biotage, Charlotte, NC, USA) and eluted with 1800 μL of hexane:isopropanol (90/10, *v*/*v*) in each well. Each eluted sample was evaporated under nitrogen and reconstituted with 300 μL of methanol:dichloromethane (65/35, *v*/*v*). Plates were shaken for 10 min and samples were transferred into high-performance liquid chromatography glass vials to be analyzed. 

High-performance liquid chromatography (HPLC)-UV analysis was performed using an Agilent 1260 liquid handling system (Agilent, Mississauga, ON, Canada) equipped with a binary pump system and a C30 reversed phase column (YMC America Inc. Allentown, PA, USA) kept at constant temperature (35 °C). Carotenoids were separated with a mobile phase consisting of methanol:water (98/2, *v*/*v*; Eluent A) and methyl-tert-butyl ether (MTBE; Eluent B; VWR, Mississauga, ON, Canada). Injection volume was set at 40μL, the flow rate was set at 1 mL/min, and the gradient elution was as follows: 2% Eluent B (initial), 2.0–80% Eluent B (0.0–27.0 min), isocratic 80% Eluent B (27.0–31.0 min), 80.0–2.0% Eluent B (31.0–31.1 min), and isocratic 2% Eluent B (31.1–34.0 min). UV detector was set at 450 nm and identification of each compound was confirmed using retention time and UV spectra (190–640 nm) of the pure compounds. Data acquisition was carried out with the Chemstation software (Agilent, Mississauga, ON, Canada). For all carotenoids the concentrations are reported in μmol/L of plasma. Value considered outlier, defined as value falling outside of the mean ±4 standard deviations (1 outlier in β-crypoxanthin), was excluded from heritability analyses.

### 2.4. Statistical Analysis

Statistical Analysis Software (SAS) was used to compute differences in CM parameters (TC, LDL-C, HDL-C, TC/HDL-C, TG, apolipoprotein B100 (apoB100), glucose, insulin, systolic blood pressure (SBP), diastolic blood pressure (DBP), and CRP) between fathers and mothers, and between daughters and sons using an unpaired *t*-test. Variables not normally distributed were log10 transformed before analyses. For heritability analysis, adjustments were made for the effects of sex, age, and categories of BMI (underweight, normal, overweight, and obese) using a standard least squares model in JMP software v12. (SAS Institute Inc. Cary, NC, USA). Categories of BMI were used to compare BMI among parents with BMI percentile among children. Cutoffs for BMI and BMI percentile were both from the World Health Organization [32,33]. Residuals from this model were used to compute heritability estimates using the variance component method implemented in QTDT v2.6.1 (Center for Statistical Genetics, Ann Arbor, MI, USA) [34]. We used a full general model in which the variance in concentration of each carotenoid was partitioned into polygenic effects (Vg), common environmental effects shared by family members (Vc), and nonshared environmental effects unique to each individual (Ve). We tested this full general model against a null model of no familial resemblance in which Vg = Vc = 0. We then computed average maximal heritability, as the proportion of variance accounted by genetic and common environmental effects ((Vg + Vc)/(Vg + Vc + Ve)), average genetic heritability, as the proportion of variance accounted by genetics effects (Vg/(Vg + Vc + Ve)), and common environmental effect, as the proportion of variance accounted by common environmental effects (Vc/(Vg + Vc + Ve)). For comparison purposes, we computed an alternative genetic model in which the variance in carotenoid concentrations was partitioned into Vg and Ve. We then computed average genetic heritability, as the proportion of variance accounted by genetic effects (Vg/(Vg + Ve)). Phenotypic Pearson correlations (ρ_P_) between seven carotenoid concentrations and 11 CM risk factors were calculated. Once again carotenoids concentrations were adjusted for the effects of sex, age, and categories of BMI. Significant phenotypic correlations (ρ_P_) between pairs of traits have been portioned into genetic (ρ_G_) and environmental (ρ_E_) correlations using a bivariate genetic analysis. Analyses were performed using SOLAR Eclipse version 7.6.4. (University of Maryland, Catonsville, MD, USA). Bonferroni corrections were used to account for multiple testing in phenotypic, genetic, and environmental correlations.

## 3. Results

### 3.1. Characteristics of Study Participants

Characteristic of study participants are presented in Table 1. Fathers and mothers had significant differences in HDL-C, TC/HDL-C, and SBP, whereas glucose concentrations were significantly different between boys and girls. Concentrations of all six carotenoids (α-carotene, β-carotene, β-cryptoxanthin, lutein, lycopene, and zeaxanthin) and total carotenoids measured in the fasting state are presented in Supplementary Table S1.

### 3.2. Heritability Analysis of Plasma Carotenoid Concentrations

Maximal heritability, genetic heritability, and common environmental effect from the full general model for the seven measurements of carotenoids are presented in Table 2. A significant familial effect was observed for lutein and lycopene (*p*-values of 6 × 10^−6^ and 4.3 × 10^−3^, respectively). Plasma lutein concentrations had a maximal heritability of 88.32%, a genetic heritability of 43.82%, and a common environmental effect of 44.50%, whereas for plasma lycopene concentrations, the maximal heritability was of 45.23%, the genetic heritability of 0%, and the common environmental effect of 45.23%.

### 3.3. Bivariate Genetic Analysis between Carotenoids and Cardiometabolic Risk Factors

Phenotypic correlations between seven measurements of carotenoids (α-carotene, β-carotene, β-cryptoxanthin, lutein, lycopene, zeaxanthin, and total carotenoids) and 11 CM risk factors (TC, LDL-C, HDL-C, TC/HDL-C, TG, apolipoprotein B100 (apoB100), glucose, insulin, SBP, DBP, and CRP) were calculated (Supplementary Table S2). Significant phenotypic correlations (*p* ≤ 0.05) were obtained for β-cryptoxanthin, lycopene, and zeaxanthin (Table 3). Positive phenotypic correlation between lycopene and DBP (*r* = 0.44) remained significant after Bonferroni correction (adjusted *p* = 0.05/11 *=* 0.0045). Phenotypic correlations were partitioned into genetic and environmental correlations using bivariate genetic analysis. Significant genetic and environmental correlations (*p* ≤ 0.05) were obtained for lycopene and β-cryptoxanthin, respectively (Table 3). Indeed, lycopene had a significant genetic correlation with DBP (*r* = 1), while β-cryptoxanthin had a significant environmental correlation with CRP (*r* = 0.61) (Table 3). Genetic and environmental correlations also remained significant after Bonferroni correction (adjusted *p* = 0.05/2 *=* 0.025). 

## 4. Discussion

As stated previously, the aim of the present study was, first, to estimate the contribution of both genetic and common environmental effects to the variance of plasma carotenoid concentrations (α-carotene, β-carotene, β-cryptoxanthin, lutein, lycopene, zeaxanthin, and total carotenoids). Familial resemblances in lutein concentrations were due to both genetic and common environmental effects, while only common environmental effects contributed to familial resemblances in lycopene concentrations. Lutein is a robust biomarker of FAV consumption [12]. Several studies, mostly based on self-reported data, also linked FAV intakes and plasma lutein concentrations [11,35,36], which is interesting considering that lutein had the strongest maximal heritability in the present study. This suggests that plasma lutein, a biomarker of consumption of FAV, is heritable. Lycopene may have potential benefits against chronic diseases such as cardiovascular diseases due to its antiatherogenic effect [13,37,38,39]. Not all studies have reported the relationship between circulating lycopene and FAV consumption [35,40,41]. Nevertheless, a considerable maximal heritability of lycopene concentrations was reported in the present study. This suggests a significant familial effect in plasma lycopene concentrations of healthy subjects, which may be related to chronic diseases. 

Farook et al. reported highly significant heritability estimates of α-carotene and β-carotene in children [21]. They used an alternative genetic model that determines which proportion of the phenotypic variance is attributable to additive genetic effects (genetic heritability). Using the same model in the present study, all genetic heritability estimates increased (Supplementary Table S3). The genetic heritability of α-carotene and β-carotene were barely significant, but lutein and lycopene still had highly significant genetic effects. The inflation in heritability estimates in the alternative genetic model may be due to the inclusion of variance due to common environmental effects with the variance due to additive genetic effects, which has been acknowledged in Farook et al. [21]. Moreover, Gueguen et al. reported a significant familial effect for serum retinol and α-tocopherol concentrations in 387 healthy French families [42]. Additive genetic and shared common environment effects for retinol (30.5% and 14.2%, respectively) and α-tocopherol were reported (22.1% and 18.7%, respectively) [42]. The results of the present study also shed light on the contribution of common environmental effect to the familial resemblances in plasma lutein and lycopene concentrations but the estimates are slightly higher than what was found by Gueguen et al. [42]. 

The aim of the present study was also, secondly, to test phenotypic, genetic, and environmental correlations between carotenoid concentrations and 11 traditional CM risk factors. There were significant phenotypic correlations between β-cryptoxanthin, lycopene, as well as zeaxanthin and CM risk factors. β-cryptoxanthin had a significant positive environmental correlation with CRP, indicating that common environmental factors affect traits in the same way (increase or decrease concentrations). Epidemiologic studies have reported an inverse correlation between β-cryptoxanthin and CRP concentrations [43,44,45,46]. However, studies including intervention trials reported positive or no association between carotenoids and CRP [47,48,49]. A systematic review and meta-analysis by Cheng et al. reported weak evidence that interventions with supplements of lycopene had an effect on plasma CRP concentrations [50]. Accordingly, a carotenoid component score accounted for only 3.3% of the total variance in serum CRP concentrations [51]. Interestingly, the relationship between β-cryptoxanthin and CRP concentrations could also be explained by TG concentrations, which are correlated with β-cryptoxanthin in the present study. Other studies have reported a correlation between CRP and TG concentrations [52,53], and a significant correlation was also observed in the present study (*r* = 0.34 *p* = 0.02). Thus, the correlation between β-cryptoxanthin and CRP concentrations may be explained through β-cryptoxanthin correlation with TG concentrations, which is concordant with the environmental correlation reported in bivariate analysis.

Moreover, lycopene had a strong significant positive genetic correlation with DBP, suggesting that shared genetic factors affecting both lycopene concentrations and DBP act in the same way. Studies have reported beneficial [54,55,56], neutral [57,58], and even detrimental [59,60] effects of lycopene on blood pressure. According to Cheng et al., there was only weak evidence that lycopene intervention had an effect on DBP [50]. According to a recent review by Thies et al., there are not enough studies to draw substantial conclusions on the effects of lycopene on blood pressure [39]. Interestingly, the strong genetic correlation suggesting shared genetic factors affecting both lycopene concentrations and DBP may be plausible since *SETD7* has been associated with both traits [15,61]. Indeed, a genetic variant (rs7680948) within *SETD7*, encoding for SET domain containing lysine methyltransferase 7, was associated with serum lycopene concentrations in Amish adults [15]. *SETD7* was also associated with DBP response and may play a role in high glucose-induced vascular dysfunction [61,62]. Genetic variants and changes in expression of genes involved in lycopene absorption and metabolism may also impact CM risk factors including blood pressure but this area still needs to be further studied [63]. 

The present study has strengths, but also some limitations. The main strength results from the study of six predominant plasma carotenoids. To the best of our knowledge, this is the first study that computed heritability and bivariate genetic analysis of so many carotenoids. The calculation of genetic and environmental correlations adds important information about the additive genetic and environmental effects that are shared between carotenoids and CM risk factors. Another strength derives from the adjustments for age, sex, and categories of BMI that were made in the heritability and correlation analyses. Several studies have reported sex differences in lutein and β-cryptoxanthin concentrations [12,64,65]. Body weight may also interfere with circulating carotenoid concentrations since these may be accumulated in adipose tissue [66,67]. Age may also impact circulating carotenoids [17,68]. Moreover, Bonferroni corrections were made to account for multiple testing. On the other hand, the study’s main limitation resides in the small sample size that limits statistical power to detect significant heritability estimates and significant correlations between traits. This may also explain why we observed a strong correlation of *r* = 1 in the bivariate analyses. The number of subjects may limit the accurate quantification of correlations between traits. The results should therefore be interpreted with caution. The study of a founder population with relatively homogeneous genetics and shared environment is a new aspect in this field [69]. However, this limits the generalization of results to other populations. Moreover, circulating carotenoids are also associated with blood lipid profile: lower TC, LDL-C, and HDL-C concentrations are associated with lower circulating carotenoids [49,70]. We did not adjust carotenoid concentrations for lipid profiles but adjustments for sex, body weight, and age may partly account for this effect since men and women depict different lipid profiles. Finally, our study did not account for diet, physical activity, smoking, and alcohol consumption of participants, all of which may affect circulating carotenoid concentrations [71,72]. Despite that we have data on diet and physical activity, we decided not to use them considering the bias related to self-reporting dietary assessment methods [6] and the fact that there is a lack of data on the link between physical activity and plasma carotenoids, especially in children [17]. Moreover, we took into account a limited number of objective confounders (age, sex, and BMI) in order to prevent the reduction of the statistical power to detect significant heritability estimates and correlations. 

In conclusion, familial resemblances in lycopene concentrations were mainly attributable to common environmental effect. Regarding lutein concentrations, familial resemblances were attributable to both genetic and common environmental effects. To the best of our knowledge, this is the first study to report on the contribution of both genetic and common environmental effects to the variance of six predominant plasma carotenoids in healthy families. Common genetic and environmental factors seem to influence carotenoids and CM risk factors but further studies are needed to better understand these possible influences and their potential impact on disease development.

## Figures and Tables

**Table 1:** Characteristic and biochemical parameters of study subjects.

Biochemical Parameters	Fathers (*n* = 6)	Mothers (*n* = 16)	Boys (*n* = 18)	Girls (*n* = 8)
Age (years)	42.0 ± 2.8	42.4 ± 6.0	11.9 ± 3.8	10.1 ± 2.0
BMI (kg/m^2^)	24.8 ± 1.3	23.5 ± 3.4	-	-
BMI percentile	-	-	48.9 ± 32.7	52.1 ± 29.8
TC (mmol/L)	4.70 ± 0.61	4.67 ± 0.55	4.35 ± 0.53	4.15 ± 0.48
HDL-C (mmol/L) ^1^	1.36 ± 0.33	1.73 ± 0.35	1.56 ± 0.28	1.54 ± 0.31
LDL-C (mmol/L)	2.83 ± 0.67	2.53 ± 0.49	2.36 ± 0.42	2.20 ± 0.52
TC/HDL-C ^1^	3.62 ± 0.88	2.79 ± 0.63	2.84 ± 0.42	2.79 ± 0.60
ApoB100 (g/L)	0.89 ± 0.22	0.77 ± 0.11	0.72 ± 0.12	0.67 ± 0.15
TG (mmol/L)	1.13 ± 0.35	0.88 ± 0.34	0.94 ± 0.39	0.89 ± 0.41
Glucose (mmol/L) ^2^	5.25 ± 0.38	5.19 ± 0.34	4.92 ± 0.24	4.73 ± 0.14
Insulin (pmol/L)	56.3 ± 8.0	70.4 ± 38.7	69.4 ± 22.5	81.0 ± 40.8
SBP (mm Hg) ^1^	117.5 ± 12.5	103.7 ± 8.9	103.2 ± 9.9	108.0 ± 11.3
DBP (mm Hg)	70.7 ± 13.6	63.3 ± 8.2	58.3 ± 8.1	65.6 ± 8.5
CRP (mg/L)	0.45 ± 0.39	0.89 ± 0.93	0.47 ± 0.79	0.33 ± 0.22

All values are means ± SD. ^1^ Means are significantly different (*p* ≤ 0.05) between fathers and mothers; ^2^ Means are significantly different (*p* ≤ 0.05) between boys and girls. Abbreviations: apolipoprotein B100 (ApoB100), C-reactive protein (CRP), diastolic blood pressure (DBP), high-density lipoprotein cholesterol (HDL-C), low-density lipoprotein cholesterol (LDL-C), systolic blood pressure (SBP), standard deviation (SD), total cholesterol (TC), total cholesterol/HDL-C (TC/HDL-C), triglycerides (TG).

**Table 2:** Heritability estimates of carotenoid concentrations.

Carotenoids	Maximal Heritability (%)	Genetic Heritability (%)	Common Env. Effect (%)	Familial Effect *p*-Value
α-carotene	43.40	28.54	14.87	0.18
β-carotene	51.29	41.68	9.61	0.14
β-cryptoxanthin	23.89	0.0043	23.88	0.49
Lutein	88.32	43.82	44.50	0.000006 *
Lycopene	45.23	0.000095	45.23	0.0043 *
Zeaxanthin	44.67	44.67	0	0.45
Total carotenoids	43.38	25.81	17.57	0.13

Heritability estimates from the full ACE general model. * Familial effect (genetic and common environmental effects) significantly different from 0 (*p* ≤ 0.05).

**Table 3:** Significant phenotypic, genetic, and environmental correlations between carotenoid concentrations and cardiometabolic risk factors.

Cardiometabolic Risk Factor	ρ_P_ ± Standard Error (SE)	*p*	ρ_G_ ± SE	*p*	ρ_E_ ± SE	*p*
**β-cryptoxanthin**						
TG	0.31 ± 0.14	0.035 *	0.35 ± 0.42	0.50	0.28 ± 0.33	0.44
CRP ^1^	0.28 ± 0.14	0.046 *	−1 ± 0	0.51	0.61 ± 0.31	0.012 **
**Lycopene**						
SBP	0.31 ± 0.14	0.042 *	0.62 ± 0.41	0.16	−0.12 ± 0.48	0.80
DBP	0.44 ± 0.12	0.0017 **	1 ± 0	0.009 **	−0.53 ± 0.55	0.23
**Zeaxanthin**						
TG	0.40 ± 0.13	0.021 *	0.61 ± 0.42	0.21	0.18 ± 0.39	0.65
Glucose	0.33 ± 0	0.011 *	0.24 ± 0.76	1	0.51 ± 0.23	0.13

Phenotypic correlation (ρ_P_) is partitioned into genetic (ρ_G_) and environmental (ρ_E_) correlations. * Significant correlation *p*-value ≤ 0.05; ** Significant Bonferroni adjusted *p*-value; ^1^ Values are log10 transformed.

## Supplementary Materials

The following are available online at http://www.mdpi.com/2072-6643/10/8/1002/s1: Table S1: Concentrations of plasma carotenoids (μmol/L of plasma); Table S2: Phenotypic correlations between carotenoid levels and cardiometabolic risk factors; Table S3: Heritability estimates of carotenoid levels.
