# Supplementary material for: Genetic and Common Environmental Contributions to Familial Resemblances in Plasma Carotenoid Concentrations in Healthy Families
Source: Nutrients. 2018 Jul 31;10(8):1002. doi: 10.3390/nu10081002 (PMC6116158; doi:10.3390/nu10081002)
Supplement: Supplementary file 1 [file nutrients-10-01002-s001.pdf]

Supplementary Table 1. Concentrations of plasma carotenoids (μmol/L of plasma).

Subjects	Lutein	Zeaxanthin	β-Cryptoxanthin	α-Carotene	β-Carotene	Lycopene	Total carotenoids
Gen01	0.36689606	0.0678172	0.48942068	0.94815424	1.96626358	1.90477202	5.74332378
Gen02	0.19040767	0.05211724	0.2671793	0.39046788	1.63413354	2.97212935	5.50643497
Gen03	0.20500873	0.04703513	0.33127708	1.3088189	1.97765103	2.14359446	6.01338535
Gen05	0.24382521	0.05322967	0.42173556	0.80394106	2.58467386	2.80014081	6.90754618
Gen06	0.21138746	0.03997405	0.47272927	0.87987086	2.33627976	2.28340529	6.2236467
Gen07	0.15361658	0.03836336	0.20938586	1.01423133	1.62869228	1.21588093	4.26017034
Gen08	0.13950888	0.03245329	0.2880089	0.56440081	1.07092427	1.52477859	3.62007474
Gen09	0.18935861	0.06023487	0.24183803	0.36889688	1.46894869	1.84769435	4.17697143
Gen10	0.45531524	0.05366406	0.68650319	1.86816097	5.09892831	1.60505358	9.76762535
Gen11	0.13301695	0.0379158	0.40775388	1.17280733	1.78868368	1.15260911	4.69278675
Gen12	0.1495655	0.03782232	0.52583487	1.20447	2.13195549	1.51354258	5.56319076
Gen13	0.22080196	0.04605841	0.40370279	1.95125405	4.36470233	1.77749248	8.76401203
Gen14	0.19439964	0.0346023	0.47240977	1.69428919	2.77308628	1.17814005	6.34692723
Gen15	0.31456871	0.07906164	0.64207259	0.585099	1.26299619	2.07400924	4.95780737
Gen16	0.20941464	0.0590733	0.66509735	0.43477479	1.2177428	1.90799437	4.49409726
Gen17	0.31659735	0.05824728	0.43726324	3.07504388	4.90271428	1.52411709	10.3139831
Gen18	0.40720388	0.06879612	0.52897983	1.77242233	2.73849116	1.40131048	6.9172038
Gen19	0.21406127	0.04334581	0.21302691	1.46073827	2.3907969	0.81416873	5.13613789
Gen20	0.38953299	0.11518446	0.54511458	0.58599723	1.94027507	1.46287634	5.03898068
Gen21	0.25823509	0.05614234	0.5515715	3.29451982	4.1048887	1.59654332	9.86190078
Gen22	0.19637546	0.05178252	0.52029086	1.43217409	2.81824867	1.43588238	6.45475399
Gen23	0.19796017	0.03400027	0.36306339	0.6257273	1.36047476	1.52337096	4.10459685
Gen24	0.23360289	0.05076413	0.42062581	1.11325177	1.89522389	1.31461194	5.02808043
Gen25	0.19012552	0.06455947	0.73073545	0.68144593	1.40988861	0.8309107	3.90766568
Gen26	0.20673794	0.04472159	0.11642864	0.1033121	0.30102258	0.5048136	1.27703645
Gen27	0.23324584	0.07133053	0.53723883	0.38361577	0.77381218	0.77033291	2.76957605
Gen28	0.22858608	0.06703344	0.40164353	0.34477048	0.57881226	0.69589933	2.31674512
Gen29	0.22569969	0.06288219	0.19197018	0.79944354	1.42605149	1.67944118	4.38548827
Gen30	0.18846852	0.05111517	0.18449405	1.76125731	3.75436001	1.36074407	7.30043914
Gen31	0.1034949	0.03639765	0.23772312	0.55324807	1.51238711	1.61846353	4.06171437
Gen32	0.1379356	0.05037577	0.58562149	1.28098545	2.66908747	1.96597652	6.6899823
Gen33	0.34863636	0.06872228	0.59045342	1.05182969	2.8442326	1.16548687	6.06936121
Gen34	0.34080966	0.08200735	0.53132439	0.87811067	3.02914672	1.32128546	6.18268426
Gen35	0.18558526	0.05637978	0.69489283	1.20488219	2.06864071	1.10169917	5.31207994
Gen36	0.21559215	0.1016761	0.82701707	2.65607443	4.85041133	1.40830847	10.0590796
Gen38	0.26706147	0.09209016	.	3.55025825	6.33803969	1.79779113	12.0452407
Gen39	0.26395015	0.06463881	0.53649644	1.52282902	3.89619121	1.72663359	8.01073921
Gen40	0.22008198	0.04334325	0.45042163	1.29773283	3.99433146	2.71061293	8.71652407
Gen41	0.33789169	0.08950376	0.40316064	2.05426427	3.12184275	1.53282075	7.53948386
Gen42	0.16596505	0.05910571	0.43107599	0.74232185	1.36939518	1.38768457	4.15554834
Gen43	0.18579005	0.05817576	0.6537702	0.9184596	2.08512821	1.28347111	5.18479494
Gen44	0.26494297	0.09297957	0.90039749	1.52573514	3.79402374	2.5744044	9.15248331
Gen45	0.49521561	0.05468624	0.35857692	1.28192912	3.42957551	1.58930011	7.2092835
Gen46	0.72653971	0.09196268	0.47041018	1.0846667	1.94897283	1.42895167	5.75150378
Gen47	0.5848228	0.06066084	0.40081868	1.18038703	2.54237772	1.59877117	6.36783824
Gen48	0.55555079	0.07599738	0.5545489	0.91123368	1.78155749	1.2136722	5.09256043
Gen49	0.21725674	0.05761664	0.4287788	0.67397551	2.02502444	1.58549025	4.98814238
Gen50	0.22004146	0.03996355	0.286557	0.66712712	1.28660567	1.18069892	3.68099372

Supplementary Table 2. Phenotypic correlations between carotenoid levels and cardiometabolic risk factors.

CM risk factor	ρP ± SE	*p*-value
	**α-carotene**	
TC	0.20 ± 0.14	0.17
LDL-C	0.092 ± 0.15	0.55
HDL-C	0.23 ± 0	0.065
TC/HDL-C	−0.10 ± 0.17	0.49
TG	0.087 ± 0.15	0.57
ApoB100	0.22 ± 0.14	0.10
Glucose	0.0094 ± 0	0.94

Insulin	−0.067 ± 0.15	0.65
SBP	−0.19 ± 0.15	0.22
DBP	−0.087 ± 0.16	0.59
CRP [1]	0.15 ± 0.14	0.30
	**β-carotene**	
TC	0.23 ± 0.14	0.14
LDL-C	0.13 ± 0.15	0.38
HDL-C	0.18 ± 0	0.15
TC/HDL-C	−0.068 ± 0.15	0.64
TG	0.017 ± 0.15	0.91
ApoB100	0.27± 0.13	0.052
Glucose	0.027 ± 0.14	0.85
Insulin	−0.15 ± 0.15	0.32
SBP	−0.25 ± 0.15	0.11
DBP	−0.048 ± 0.16	0.77
CRP [1]	0.12 ± 0.14	0.42
	**β-cryptoxanthin**	
TC	−0.094 ± 0.15	0.53
LDL-C	−0.22 ± 0.15	0.15
HDL-C	0.027 ± 0.15	0.86
TC/HDL-C	−0.092 ± 0.15	0.54
TG	0.31 ± 0.14	0.035*
ApoB100	−0.041 ± 0.15	0.79
Glucose	0.12± 0.14	0.41
Insulin	0.18 ± 0.14	0.22
SBP	−0.017 ± 0.16	0.91
DBP	0.057 ± 0.16	0.71
CRP [1]	0.28 ± 0.14	0.046*
	**Lutein**	
TC	0.19 ± 0.15	0.22
LDL-C	0.083 ± 0.15	0.58
HDL-C	0.26 ± 0.17	0.077
TC/HDL-C	−0.17 ± 0.15	0.25
TG	−0.015 ± 0.15	0.92
ApoB100	0.14 ± 0.14	0.32
Glucose	0.095 ± 0.14	0.51
Insulin	−0.16 ± 0.15	0.30
SBP	−0.26± 0.15	0.092
DBP	−0.23 ± 0.15	0.14
CRP [1]	0.00038 ± 0.14	1.00
	**Lycopene**	
TC	0.16 ± 0.15	0.30
LDL-C	0.11 ± 0.16	0.48
HDL-C	-	-
TC/HDL-C	−0.11 ± 0.14	0.44
TG	0.012 ± 0.15	0.94
ApoB100	0.12 ± 0.15	0.43
Glucose	0.12 ± 0.14	0.41
Insulin	0.052 ± 0.15	0.73
SBP	0.31 ± 0.14	0.042*
DBP	0.44 ± 0.12	0.0017**
CRP [1]	−0.024 ± 0.14	0.87
	**Zeaxanthin**	
TC	0.17 ± 0.16	0.29
LDL-C	0.092 ± 0.16	0.56
HDL-C	0.042 ± 0.15	0.78
TC/HDL-C	0.048 ± 0.15	0.75
TG	0.40 ± 0.13	0.021*
ApoB100	0.22 ± 0.14	0.13
Glucose	0.33 ± 0	0.011*
Insulin	0.054 ± 0.15	0.72
SBP	0.042 ± 0.16	0.79
DBP	0.074 ± 0.16	0.65
CRP [1]	0.089 ± 0.15	0.55
	**Total carotenoids**	

TC	0.24 ± 0.14	0.12
LDL-C	0.12 ± 0.15	0.43
HDL-C	0.21 ± 0.14	0.14
TC/HDL-C	−0.094 ± 0.14	0.52
TG	0.079 ± 0.15	0.60
ApoB100	0.27 ± 0.13	0.058
Glucose	0.058 ± 0.14	0.69
Insulin	−0.091 ± 0.15	0.54
SBP	−0.14 ± 0.15	0.35
DBP	0.037 ± 0.16	0.82
CRP [1]	0.14 ± 0.14	0.28

* Significant correlation *p*-value ≤ 0.05. ** Significant Bonferroni adjusted *p*-value (0.05/11 = 0.0045).[1] Values are log10 transformed. Abbreviations: Apolipoprotein B100 (ApoB100), Cardiometabolic (CM), C-reactive protein (CRP), Diastolic blood pressure (DBP), High-density lipoprotein cholesterol (HDL-C), Low-density lipoprotein cholesterol (LDL-C), Phenotypic correlation (ϱ_P), Standard error (SE), Systolic blood pressure (SBP), Total cholesterol (TC), Total cholesterol/HDL-C (TC/HDL-C), Triglycerides (TG).

Supplementary Table 3. Heritability estimates of carotenoid levels.

Carotenoids	Genetic heritability (%)	Genetic effect *p*-value
α-carotene	48.38	0.0793
β-carotene	56.23	0.0504
β-cryptoxanthin	31.29	0.3578
Lutein	99.81	0.000009 *
Lycopene	76.61	0.0023 *
Zeaxanthin	44.65	0.208
Total carotenoids	51.68	0.0541

Heritability estimates from the alternative AE model. * Genetic effect significantly different from 0 ($p \le 0.05$).
